# Green Spectrophotometric Determination of Organophosphate in Selected Fruits and Vegetables

**DOI:** 10.1155/2023/6691659

**Published:** 2023-06-08

**Authors:** Hemraj Sharma, Hari Prasad Sapkota, Kanchan Paudel, Anusha Raila, Sujata Kandel, Pramod Chaudhary, Kritika Bhattarai

**Affiliations:** ^1^Department of Pharmacy, Shree Medical and Technical College, Bharatpur, Chitwan, Nepal; ^2^Department of Pharmacy, Rapti Technical School, Rapti, Dang, Nepal; ^3^Nepal Medical College Teaching Hospital, Jorpati, Kathmadu, Nepal

## Abstract

A simple, sensitive, precise, and environmentally safe spectrophotometry method was developed and validated for the determination of organophosphate in various fruits and vegetables using a UV-Visible spectrophotometer using a magnesia mixture. The volume of reagent used for analysis and the stability of the color complex were also optimized. The drug showed a stable white color complex at 420 nm. The greenness of the methods was estimated using an ecoscale (84), the Green Analytical Procedure Index, and AGREE (0.89), which were found to be excellent green method based on spectrophotometric determination. The method was validated using ICH guidelines and has acceptable values for linearity (0.5–2.5 mg/ml), accuracy (98.5–102.5%), precision, robustness, limit of detection (0.16 mg), and limit of quantification (0.486 mg). The concentration of the organophosphate in the analyzed sample was in the range of 0.003 to 2.45 mg. Altogether, the proposed green analytical method was found to be a simple, selective, sensitive, accurate, and ecofriendly method for the analysis of organophosphate in various fruits and vegetables.

## 1. Introduction

Pesticides are chemical substances or mixture of chemicals meant for preventing, repelling, destroying, or controlling any pest like insects or other organisms. As per WHO 2008, pesticide poisoning is any pesticide-related injury or health effect including systemic and nonsystemic effect, resulting from suspected or confirmed exposure to a pesticide [1]. These are synthetic molecules aimed at being toxic towards fungi, plants, or animals that are detrimental to cultures. Fungicides, herbicides, and insecticides have been developed in order to control as specifically as possible these pests in order to protect cultures. Nevertheless, these pesticides can be toxic to human and wild fauna [2]. Vegetable cultivation attracts high rates of the application of pesticides and farmers in many developing countries use many acutely toxic insecticides on those crops. Apart from application of pesticides exposures can also occur for farmers involved in the harvesting process or who enter the sprayed field too soon after spraying [3]. Farmers, particularly in areas of commercial vegetable production, are primarily relying on chemical pest control methods. The frequency of pesticides used is 3 times per week. Around 97.6% of subjects used incomplete personal protective equipment [4].

Most of the Nepalese population lives in rural areas, and nearly 80% of the country's people are involved in agriculture which is an important factor for the national economy. Cultivation of vegetables in the agricultural lands of Nepal normally generates high earnings per unit area in comparison to the crops although it is cultivated in a wide area of Nepalese agricultural lands. The number of small-scale farmers growing vegetables for the domestic market in Nepal is increasing. Pesticides are widely used in Nepal to control various pests and disease in agriculture as well as livestock production. The increasing demands of food and vegetables have led to an increased use of pesticides. Among the pesticides used in Nepal, organophosphate compounds are the most commonly used [3].

In the 1930s, organophosphates were used as insecticides, but the German military developed these substances as neurotoxins in the Second World War [5]. Organophosphates (OP) are chemical substances produced by the process of esterification between phosphoric acid and alcohol. These chemicals are the main components of herbicides, pesticides, and insecticides [6]. Organophosphate poisoning can be acute or chronic. The symptoms of acute toxicity are hypersecretion, bronchoconstriction, myosis, diarrhea, bradycardia, central nervous system (CNS) depression, seizure, cyanosis, and coma [7]. The organophosphate poisoning was experienced by farmers through inhalation and dermal contact. The severity depends on pesticide type, dose, and duration of application and frequency of application. The intensity of organophosphate poisoning was influenced by the area of pesticide application, climate skill of the application, and personal [8]. The mechanism of organophosphate`s toxicity is via inhibiting the acetylcholinesterase (AchE), is an enzyme that degrades the neurotransmitter acetylcholine to choline and acetic acid. The inhibition of AchE will cause the increase in acetylcholine concentration in the synapse. This mechanism will cause some nicotinic and muscarinic symptoms and central and peripheral nervous system toxicity [9].

Due to the excess demand of numbers of fruits and vegetables, Nepal depends a lot in India which supplies these food stuffs from a long decade. There were few news stories in the newspaper, TV, and so on. related to use of excess pesticides in fruits and vegetables which are transported in border area of Nepal from India. Hence, this study will definitely provide the scientific statement regarding this issue. Among various fruits available in the markets, the selection of fruits i.e. mango and watermelon is to rationalize the study as these fruits are mostly available and consumed fruits in our Nepalese society. These are consumed in every family visit and devour by children, so studying these can make our research more relevant. The reason for choosing other fruits like kiwi is the popularity in the current context of Nepal. Kiwi and lemon are now famous as a source of Vitamin C, support heart health and the digestive system, protect anemia, reduce cancer risk, and to boost immune system. There is a rising demand for organic agricultural products due to consumer concern about the strong contamination of vegetables from the applied pesticides. Therefore, it is essential to identify the presence of pesticide residue in vegetables so that consumers could be protected. In this regard, this study is going to be initiated to assess the present status of organophosphate pesticide residue in eggplant, tomato, cucumber, cabbage, ladyfinger, cowpea, pointed gourd, and bean.

Various research studies have conducted for the determination of the organophosphate in foods using methods such as P-NMR [10], conductometry [11], and GC-MS/GC-NPD [12], where most of the methods use HPLC [13–16] or liquid-mass spectrometry [17]. Since these methods are highly sophisticated, expensive, and use a large amount of organic solvents along with a high amount of instrumental energy, so a developing country such as Nepal could not afford the availability of such machines in every local area. The use of less sophisticated analytical methods like UV-Visible spectrophotometer with more concern on greenness profile, if provides the similar results like above-mentioned methods, this will be an extremely economical method of detection. Hence, a simple, green economic UV-Visible spectrophotometric method was developed and validated for the analysis of fruits and vegetables.

## 2. Materials and Methods

### 2.1. Materials

#### 2.1.1. Plant Material

The fruits and vegetables (mango, kiwi, lemon, watermelon, tomato, cowpea, cucumber, cabbage, pointed gourd, ladyfinger, and eggplant) were collected from the commercial market of Raxaul, India, and local market from Chitwan.

#### 2.1.2. Pure Sample and Chemicals

Ethanol 95% (Changshu Hongsheng Fine Chemical Co. Ltd.), bromine water 99% (Nike Chemical India), disodium hydrogen orthophosphate 99.5% (Merck Life Science Pvt. Ltd.), ammonium chloride 99.7% (Sisco Research Laboratories Pvt. Ltd.), and magnesium chloride 99.0–101.0% (Fisher Scientific) were used for this research.

#### 2.1.3. Equipment and Software Required

LT-2100 double beam UV-visual spectrophotometer with a 10 mm quartz cuvette was used to record the absorbance. The greenness of the method was established using GAPI chart version 1.0.0.

### 2.2. Methodology

#### 2.2.1. Collection of Samples

The fruits and vegetables samples were collected from the local market (Chitwan) as well as from Raxaul, India, and proceed for extraction.

#### 2.2.2. Extraction

**Figure 1:**
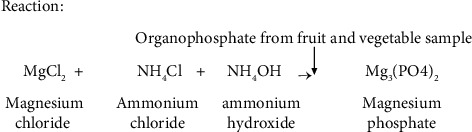
Formation of color complex.

A stainless steel knife was used to cut fruits and vegetable samples, sliced and diced them, and 1 gm sample was placed inside a glass test tube, as shown in the Figure 1. The extraction was carried as per Knowledge-Based Integrated Sustainable Agriculture and Nutrition (KISAN) II Project [18] using 2 ml of 95% alcohol (ethanol) and bromine water solution. Finally, the extracted solution was poured into a clean test tube prior to proceeding for test.

### 2.3. Method Development

Method development was done following the article published by Kumar Reddy et al. [19].

#### 2.3.1. Selection of Suitable Wavelength

The wavelength maximum was selected by varying the wavelength from 200 to 800 nm.

#### 2.3.2. Selection of Reagent Volume

The volume of optimization was carried out by varying the volume from 1 to 3 ml.

#### 2.3.3. Mechanism of Color Complex Formation

Magnesia mixture was prepared by adding 2 gm magnesium chloride solution to 1 gm ammonium chloride and adding about 30 drops of ammonium hydroxide to above-mentioned solution after boiling and cooling it till the strong smell of ammonia is obtained. Thus, prepared solution is allowed to react with the sample extract to obtain white color of the magnesium phosphate and detected in UV-Visible spectrophotometer.

The intensity of the white precipitate depends on the amount of magnesium phosphate, which in turn depends on the phosphate present in fruit and vegetable samples. This is the basis of organophosphate pesticide analysis.

### 2.4. Sample Preparation

From each of the extracts, 1 ml sample was taken and added with the 2 ml of magnesia mixture in 10 ml of volumetric flask. The final volume is made up by distilled water for both Indian and local samples.

## 3. Results and Discussion

### 3.1. Selection of Wavelength Maxima

Disodium orthophosphate was taken as the standard for this analysis. It was allowed to react with the magnesia mixture to form a white color complex. The absorbance was observed at the maximum wavelength 420 nm after varying wavelength from 200  to  800 nm.

### 3.2. Optimization of Volume of Reagent

The optimization of the reagent was first established by varying volume of the reagent (1 ml to 3 ml), where the maximum absorbance at 2 ml was found. Hence, it was selected after optimizing volume of reagent, as shown in Table 1.

### 3.3. Stability of Colored Complex

After optimizing the volume of the reagent it was subjected for the stability test. The optimum time for completion of the reaction between disodium orthophosphate and magnesia mixture to obtain the white color was 1 min, and the complex was stable for 1 hour. Then the absorbance was measured, and it was quite stable with precise measurement which is shown in Table 2.

### 3.4. Preparation of Calibration Curve

0.5 gm of disodium orthophosphate was weighed and dissolved in distilled water and made up to 100 ml to prepare a 5 mg concentration of solution. The secondary stock solution was prepared at 0.5 mg, 1 mg, 1.5 mg, 2 mg, and 2.5 mg concentrations, respectively, after the addition of magnesia mixture and dilution with water. Finally, absorbance was observed in a UV spectrophotometer at a wavelength of 240 nm after obtaining it as the maximum wavelength.

### 3.5. Determination of Concentration of Organophosphate in Samples

#### 3.5.1. Analysis in Vegetables

**Figure 2:**
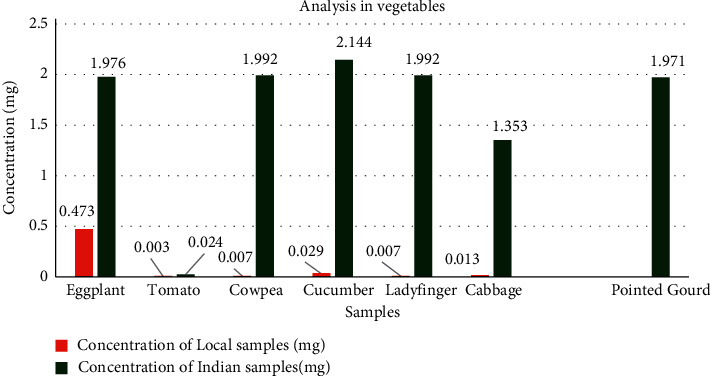
Comparative analysis of organophosphate in vegetables.

All randomly selected samples were collected and extraction of it was carried out. 1 ml of the extract was taken and diluted to 10 ml volumetric flask with 2 ml of magnesia mixture and 7 ml of water. The mixture was observed in a UV-Visible spectrophotometer and the absorbance obtained was recorded. Finally, the organophosphate concentration on each vegetable was calculated and compared, as shown in Figure 2 and a statistical analysis was established (Table 3).

#### 3.5.2. Analysis in Fruits

**Figure 3:**
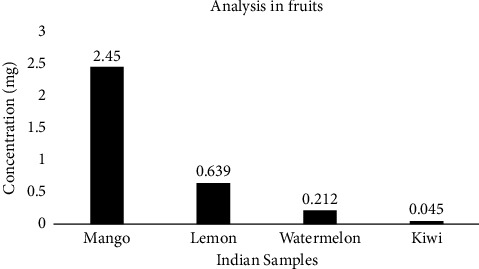
Analysis of organophosphate in fruits.

Due to the unavailability of local seasonal fruit samples during the time of research, only Indian fruit samples were used for the determination of organophosphate, as shown in Figure 3.

### 3.6. Validation

The method was validated as per International Conference on Harmonization (ICH) Guidelines [20].

#### 3.6.1. Linearity

**Figure 4:**
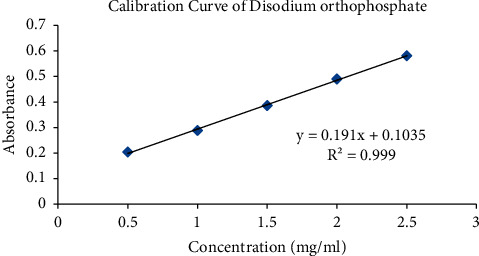
Calibration curve of disodium orthophosphate.

The absorbance of complex was analyzed using UV-Visible spectrophotometer. The linearity graph is shown as per Figure 4.

#### 3.6.2. Limit of Detection and Quantification

The limit of detection (LOD) and limit of quantification (LOQ) for the procedure were performed and the data were obtained as 0.160 mg and 0.486 mg, respectively.

#### 3.6.3. Precision

The data for intraday and interday precision studies were obtained from three different concentrations. The percentage RSD was calculated and shown in the Table 4. The % RSD should be less than 2.5%.

#### 3.6.4. Accuracy

The accuracy of the method was evaluated in triplicate at three concentration levels i.e. 80%, 100%, and 120%. The percentage of recoveries was calculated and shown in Table 5.

#### 3.6.5. Robustness

*(1) Variation of Wavelength*. The robustness of the sample was carried out by the variation of wavelength at 239 and 241 nm (Table 6). The concentration of the sample selected was 1 mg/ml. The % RSD should be less than 2.5%, which showed that variation in the wavelength showed the method to be not robust.

*(2) Variation of Reagent Volume*. The robustness of the sample was carried out by variation of reagent volume i.e. 1.8 ml and 2.2 ml, as shown in Table 7. The concentration of the sample selected was 1 mg/ml. The % RSD should be less than 2.5%, which showed that variation in the reagent volume showed the method to be not robust.

### 3.7. Greenness Profile Evaluation of the Proposed Spectrophotometric Method

#### 3.7.1. Assessment Using Analytical Ecoscale

An excellent semiquantitative method applied to assess the greenness profile of the analytical methods is the analytical ecoscale [21, 22]. Based on the penalty points, the total score of the method is calculated. The ideal green analytical method is with an ecoscale score of 100.75 and 50 are the green methods which are named as excellent and fair green analytical methods, respectively. If the penalty point is less than 50, it is called the deficient green method. The ecoscale score of the proposed green analytical method is 84, as shown in Table 8.

#### 3.7.2. Assessment Using Green Analytical Procedure Index (GAPI)

The qualitative method meant to measure the greenness is GAPI, which calculate greenness based on the stages involved in an analytical method [23, 24]. The two main stages of GAPI are sample preparation and instrumental assessment. A pictogram of five pentagrams is a visual output in GAPI and is used to evaluate and quantify the low, medium, and high environmental impact involved for each step of the methodology which can be analyzed by the green, yellow, or red color in each pentagram.

**Figure 5:**
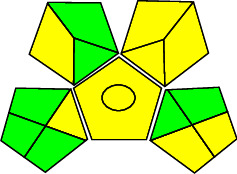
Assessment of proposed method by GAPI.

The application of GAPI in the proposed method is given in Table 9, and the pictogram is represented in Figure 5, which shows the method, has satisfied most of the criteria and confirms the proposed method as ecofriendly.

#### 3.7.3. Assessment Using Analytical Greenness Metric (AGREE)

**Figure 6:**
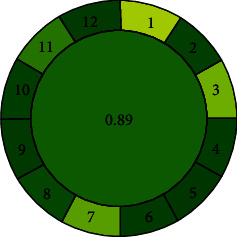
Assessment of proposed method by AGREE.

AGREE depends on 12 parameters equal to the 12 principles of Green Analytical Chemistry. Each principle or parameter contains a score range 0-1, which is calculated based on the hazardous to a particular principal of greenness. It looks like a classical clock shape consisting of numbers 1–12 on the edge of the circle, representing the philosophy of 12 principles [25]. As shown in Figure 6 with 0.89, the proposed method indicates the method was greenest in all aspects of green principles.

## 4. Conclusion

A novel green spectrophotometric method was designed using a magnesia mixture for the analysis of organophosphates in fruits and vegetables. Direct analysis of organophosphates was not possible due to a lack of chromophore which was resolved by a simple derivatization method using magnesia mixture. Validation of the proposed method was carried out as per ICH guidelines, allowing application of the proposed method in the determination of the fruits and vegetables samples. Ecoscale, GAPI, and AGREE assessment methods also affirmed the ecosafety of the developed spectrophotometric method and can be adapted to the established quality of other fruits and vegetables for organophosphate analysis.

## Figures and Tables

**Table 1:** Optimization of volume of reagent.

Volume of reagent (ml)	Absorbance
1	0.115
1.5	0.718
**2**	**0.836**
2.5	0.720
3	0.831

**Table 2:** Stability of colored complex.

Time (minute)	Absorbance
10	0.857
20	0.854
30	0.818
40	0.818
50	0.809
60	0.802
70	0.772

**Table 3:** Statistical analysis of the data.

Concentration of Indian sample in mg (*X*_1_)	Concentration of local sample in mg (*X*_2_)	*X*_1_^2^	*X*_2_^2^
1.976	0.473	3.904576	0.223729
0.024	0.003	0.000576	0.000009
1.992	0.007	3.968064	0.000049
2.144	0.029	4.596736	0.000841
1.992	0.007	3.968064	0.000049
1.353	0.013	1.830609	0.000169
∑*X*_1_ = 9.481	∑*X*_2_ = 0.532	∑*X*_1_^2^ = 18.26863	∑ *X*_2_^2^ = 0.224846

*S*_p_^2^ = 1/(*n*_1_ + *n*_2_ − 2) [∑*X*_1_^2^ − (∑*X*_1)_^2^/*n*_1_ + ∑*X*_2_^2^ − (∑*X*_2_)^2^/*n*_2_]. *S*_p_^2^ = 0.346. Again, mean of first sample = ∑*X*_1_/*n*_1_ = 1.58. Mean of second sample = ∑*X*_2_/*n*_2_ = 0.00887. Similarly, independent *t*-test (*t*) = (mean of 1^st^ sample − mean of 2^nd^ sample)/√ (*S*_p_^2^/(1/*n*_1_ + 1/*n*_2_) = 4.391244267. Here, d.*f* = *n*_1_+*n*_2_ − 2 = 6+6 − 2 = 10. The tabulated value of *t*-test at 5% level of significance at 10 d. *f*. is 2.228 (two tails). Now, *p* value for the *t*-test is between 0.002 − 0.001. Let's call it 0.0015. So, *p* value is lower than 0.05. Thus, calculated value > tabulated value so there is significant greater amount of organophosphate in Indian sample than local sample. Also, the *p* value shows significant difference in concentration.

**Table 4:** Precision result.

Drugs	Amount (mg)	Intraday (*n* = 3) (mean ± SD)	% RSD	Interday (*n* = 3) (mean ± SD)	% RSD	Acceptance criteria % RSD
Disodium orthophosphate	1	0.287 ± 0.003	1.066	0.286 ± 0.0030	1.066	<2.5%
	1	0.177 ± 0.002	1.423	0.048 ± 0.001	2.0	
	1	0.1087 ± 0.001	1.407	0.015 ± 0.0005	3.9	

**Table 5:** Accuracy result.

Drug	Amount (mg)	Recovery level (%)	Amount added	Recovery amount	Amount recovered	% recovery	Acceptance criteria %
Disodium hydrogen orthophosphate	2	80	0.8	1.78	1.81	99.33	98–102
			0.8	1.81		100.78	
			0.8	1.84		101.94	
	2	100	1	2.05	2.16	102.5	98–102
			1	2.02		101	
			1	1.97		98.5	
	2	120	1.2	2.23	2.22	101.49	98–102
			1.2	2.21		100.3	
			1.2	2.23		101.49	

**Table 6:** Variation of wavelength for robustness.

Wavelength (nm)	Absorbance			% RSD
	*A*1	*A*2	*A*3	
239	0.199	0.201	0.196	1.267
241	0.209	0.211	0.199	3.1

**Table 7:** Variation of reagent volume for robustness.

Volume of reagent (ml)	Absorbance			% RSD
	*A*1	*A*2	*A*3	
1.8	0.176	0.181	0.174	2.04
2.2	0.126	0.122	0.130	3.17

**Table 8:** Penalty points for the determination of organophosphates by proposed spectrophotometric method.

Reagent/instruments	Penalty points
	Proposed spectrophotometric method
Ethanol	4
Bromine water	4
Disodium hydrogen orthophosphate	0
Ammonium chloride	1
Magnesium chloride	1
Distilled water	0
Occupational hazards	3 (because of bromine water)
Waste	3
Instruments energy	0
Total penalty points	Σ100−16 = 84
Analytical ecoscale total score	84
Comment	Excellent green analytical method

**Table 9:** Assessment of GAPI for the proposed method.

S. N		Category	Proposed method
*I*	*Sample preparation*		
1	Collection	UV	Green
2	Preservation	None	Green
3	Transport	None	Green
4	Storage	None	Green
5	Type of method: Direct or indirect	Simple procedures	Yellow
6	Scale of extraction	Simple extraction using ethanol	Green
7	Solvents/reagents used	Green solvents	Yellow
8	Additional treatments	None	Green
9	Reagent and solvents amount	<10 mL	Green
10	Health hazard	None	Green
11	Safety hazard	Bromine water was used very less so that flammability will be negligible	Green

*II*	*Instrumentation*		
12	Energy	UV consumes ≤0.1 kWh per sample	Green
13	Occupational hazard (OH)	None	Green
14	Waste	Waste generated by the proposed method was 1–10 mL	Yellow
15	Waste treatment	Low degradation	Yellow

Additional mark: quantification ring in the middle of GAPI: procedure for quantification.

## Data Availability

The data used to support the findings of this study are included within the article.

## References

[1] Joshi S. K. (2003). Pesticides poisoning in Nepal. *Kathmandu University Medical Journal*.

[2] Combarnous Y. (2017). Endocrine Disruptor Compounds (EDCs) and agriculture: the case of pesticides. *Comptes Rendus Biologies*.

[3] Dinham B. (2003). Growing vegetables in developing countries for local urban populations and export markets: problems confronting small-scale producers. *Pest Management Science*.

[4] Diwakar J., Prasai T., Pant S. R., Jayana B. L. (1970). Study on major pesticides and fertilizers used in Nepal. *Scientific World*.

[5] A Ghorab M., Khalil M. S. (2015). Toxicological effects of organophosphates pesticides. *International Journal of Environmental Monitoring and Analysis*.

[6] Chauhan A., Arora J., Ranjan A. (2022). Advances in characterization of probiotics and challenges in industrial application. *Biotechnology & Genetic Engineering Reviews*.

[7] Thundiyil J. G., Stober J., Besbelli N., Pronczuk J. (2008). Acute pesticide poisoning: a proposed classification tool. *Bulletin of the World Health Organization*.

[8] Jors E., Morant R. C., Aguilar G. C. (2006). Occupational pesticide intoxications among farmers in Bolivia, a cross-sectional study. *Environmental Health*.

[9] Namba T., Nolte C. T., Jackarel J. (1971). Poisoning due to organophosphate insecticides. Acute and chronic manifestation. *Americas Journal of Medicine*.

[10] Talebpour Z., Ghassempour A., Zendehzaban M., Bijanzadeh H. R., Mirjalili M. H. (2006). Monitoring of the insecticide trichlorfon by phosphorus-31 nuclear magnetic resonance (31P NMR) spectroscopy. *Analytica Chimica Acta*.

[11] Zehani N., Kherrat R., Dzyadevych S. V., Jaffrezic-Renault N. (2015). A microconductometric biosensor based on lipase extracted from Candida rugosafor direct and rapid detection of organophosphate pesticides. *International Journal of Environmental Analytical Chemistry*.

[12] Srivastava A. K., Rai S., Srivastava M. K., Lohani M., Mudiam M. K., Srivastava L. P. (2014). Determination of 17 organophosphate pesticide residues in mango by modified QuEChERS extraction method using GC-NPD/GC-MS and hazard index estimation in Lucknow, India. *PLoS One*.

[13] Baig S. A., Akhtera N. A., Ashfaq M., Asi M. R. (2009). *Determination of the Organophosphorus Pesticide in Vegetables by HPLC*.

[14] Ioerger B. P., Smith J. S. (1993). Multiresidue method for the extraction and detection of organophosphate pesticides and their primary and secondary metabolites from beef tissue using HPLC. *Journal of Agricultural and Food Chemistry*.

[15] Mahajan R., Chatterjee S. (2018). A simple HPLC–DAD method for simultaneous detection of two organophosphates, profenofos and fenthion, and validation by soil microcosm experiment. *Environmental Monitoring and Assessment*.

[16] Brayan J. G., Haddad P. R., Sharp G. J., Dilli S., Desmarchelier J. M. (1988). Determination of organophosphate pesticides and carbaryl on paddy rice by reversed-phase high-performance liquid chromatography. *Journal of Chromatography A*.

[17] Mol H. G. J., van Dam R. C., Steijger O. M. (2003). Determination of polar organophosphorus pesticides in vegetables and fruits using liquid chromatography with tandem mass spectrometry: selection of extraction solvent. *Journal of Chromatography A*.

[18] International W. (2014). *Knowledge -based Integrated Sustainable Agriculture and Nutrition (KISAN) Project Pesticides Evaluation Report and Safer Use Action Plan (PERSUAP)*.

[19] Kumar Reddy K. P., Prathap K. M. S., Sharma H., Kumar K. V. (2019). A simple colorimetric method for the determination of raloxifene hydrochloride in pharmaceuticals using modified romini’s reagent. *International Journal of Analytical Chemistry*.

[20] ICH Validation of analytical procedures: text and methodology Q2 (R1).

[21] Sharma H., Sharma A., Sharma B., Karna S. (2022). Green analytical approach for the determination of zinc in pharmaceutical product using natural reagent. *International. Journal of Analytical Chemistry*.

[22] Van Aken K., Strekowski L., Patiny L. (2006). Eco-scale, a semi quantitative tool to select an organic preparation based on economical and ecological parameters. *Beilstein Journal of Organic Chemistry*.

[23] Płotka-Wasylka J. (2018). A new tool for the evaluation of the analytical procedure: green Analytical Procedure Index. *Talanta*.

[24] Kannaiah K. P., Sugumaran A., Chanduluru H. K., Rathinam S. (2021). Environmental impact of greenness assessment tools in liquid chromatography—a review. *Microchemical Journal*.

[25] Vuddagiri M. N., Boddu V. (2021). A new approach for evolution and quantification of Triamcinolone acetonide in medication shots by using RP-HPLC. *Journal of Applied Pharmaceutical Science*.

